# Connection between MHC class II binding and aggregation propensity: The antigenic peptide 10 of *Paracoccidioides brasiliensis* as a benchmark study

**DOI:** 10.1016/j.csbj.2023.02.031

**Published:** 2023-02-18

**Authors:** Rodrigo Ochoa, Thyago R. Cardim-Pires, Ricardo Sant’Anna, Pilar Cossio, Debora Foguel

**Affiliations:** a Biophysics of Tropical Diseases, Max Planck Tandem Group, University of Antioquia, 050010 Medellin, Colombia; b Medicinal Chemistry, Boehringer Ingelheim Pharma GmbH & Co KG, 88397 Biberach/Riss, Germany; c Instituto de Bioquímica Médica Leopoldo de Meis, Universidade Federal do Rio de Janeiro, Rio de Janeiro, Brazil; d Center for Computational Mathematics, Flatiron Institute, New York, NY, United States; e Center for Computational Biology, Flatiron Institute, New York, NY, United States

**Keywords:** MHC class II, *Paracoccidioides brasiliensis*, Binding, Aggregation propensity, Amyloid fibril, Peptide Design, Molecular Dynamics

## Abstract

The aggregation of epitopes that are also able to bind major histocompatibility complex (MHC) alleles raises questions around the potential connection between the formation of epitope aggregates and their affinities to MHC receptors. We first performed a general bioinformatic assessment over a public dataset of MHC class II epitopes, finding that higher experimental binding correlates with higher aggregation-propensity predictors. We then focused on the case of P10, an epitope used as a vaccine candidate against *Paracoccidioides brasiliensis* that aggregates into amyloid fibrils. We used a computational protocol to design variants of the P10 epitope to study the connection between the binding stabilities towards human MHC class II alleles and their aggregation propensities. The binding of the designed variants was tested experimentally, as well as their aggregation capacity. High-affinity MHC class II binders *in vitro* were more disposed to aggregate forming amyloid fibrils capable of binding Thioflavin T and congo red, while low affinity MHC class II binders remained soluble or formed rare amorphous aggregates. This study shows a possible connection between the aggregation propensity of an epitope and its affinity for the MHC class II cleft.

## Graphical Abstract

**Figure:**
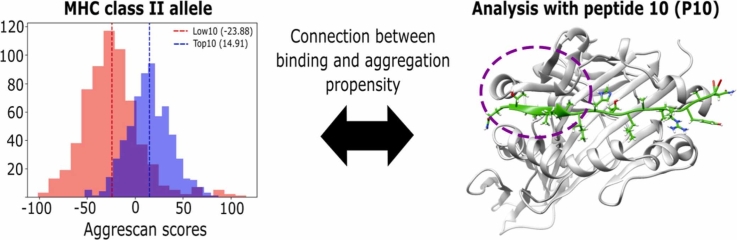

## 1 Introduction

The Major Histocompatibility Complex class II (MHC-II) is a key receptor involved in the human adaptive immune response [1]. MHC-II is a promiscuous peptide binder having affinities for a diverse set of peptides, as shown in the massive public databases [2], [3], [4]. A key aspect of the binding process is the peptide-bound stability, which assesses the time the peptide is bound to the MHC-II cleft. One expects that high-affinity and high-stability binding peptides to MHC-II are crucial to improve immune responses, and consequently enhance the effect of immunotherapies [5]. But what are the determinant biophysical and structural properties of peptides to tightly bind to MHC-II?

The prediction of MHC-II binders is a common task in epitope prediction algorithms and the design of potential vaccines [6], [7]. Most available protocols rely on the sequence-based identification of motifs in antigen regions, supported by machine learning models able to filter epitopes of different sizes by predicted binding affinities [2]. Other research has focused on structural information of peptide/MHC-II complexes (pMHC-II) that study the effect of the peptide conformations in the binding process [8], [9]. Known properties of MHC-II peptide binders are the presence of neutral amino acids interacting with key binding pocket positions [3], the formation of a bound polyproline-like II secondary structure in the MHC-II cleft [10], [11], and the importance of the peptide binding stability to trigger subsequent immunological effects [12].

In addition to peptide binding affinity, other properties to evaluate are their solubility and aggregation propensity. In principle, protein aggregation can hamper different processes related to proteins/peptides usage, hence, design strategies that control those characteristics are essential to further immunotherapy optimization [13], [14]. Many proteins and peptides in solution can aggregate into amyloid fibrils (AF), a type of aggregate that can accumulate in organs and tissues causing diseases such as Parkinson’s and Alzheimer’s [15]. This is the case of P10 (QTLIAIHTLAIRYAN) [16], a 15-mer epitope derived from the glycoprotein 43 (gp43) of *Paracoccidioides brasiliensis* (Pb), a dimorphic fungus that lives in the ground and can infect the host by inhalation [17]. Interestingly, this peptide is also a highly binding epitope of MHC-II in the nanomolar (nM) range toward a set of MHC-II alleles [18]. The epitope is found in the region 181–195 of gp43 and has been extensively studied *in vitro* and *in vivo*, protecting mice against Pb infection as effectively as using the full gp43 for immunization [19]. In 2021, it was reported that in solution P10 can aggregate into insoluble AF. The formation of P10-composed AF depends on the peptide concentration, pH and temperature. AF seeds (fragments of mature AF) of P10 were able to induce the aggregation of another gp43-derived peptide used as an immunogenic agent [20].

Here, we study the correlation between MHC-II peptide binding and aggregation propensity using P10 as a benchmark system. We first performed a bioinformatic assessment of the Immune Epitope Database (IEDB) to relate MHC-II affinity to aggregation *via* computational predictors, finding that high-affinity peptides have a large aggregation propensity. Then, we used the PARCE protocol [21] to design new variants of the P10 peptide with diverse binding affinities for the human MHC-II allele DRB1*01:01, providing a range of affinities to relate with aggregation. After implementing different design strategies, we obtained a list of 18 candidate peptides that were tested experimentally with the Proimmune REVEAL® binding assay. For 11 of these, including P10, we evaluated their aggregation capacity *in vitro* by using Thioflavin (ThT) and Congo red binding assays and transmission electron microscopy (TEM). Our findings suggest that a peptide's tendency to aggregate into amyloid fibril might modulate the binding affinities to MHC-II.

## 2 Methods

### 2.1 Aggregation propensity and binding to MHC class II

To overview potential aggregation propensities for MHC-II peptides, we used a set of peptides having approximately 44,000 experimental binding affinity endpoints, available at the Immune Epitope Database (IEDB) [2]. We selected four MHC-II alleles: DRB1*01:01, 03:01, 04:01 and 15:01. For each allele, we used the most populated cluster composed of 15-mer peptides with a wide range of IC_50_ values. We created two sets: the top binders (top 10 % ranked with the IC_50_ - Top10) and lowest affinity binders (lowest 10 % - Low10). The number of peptides included for both sets per allele were 1,256 for 01:01, 284 for 03:01, 328 for 04:01, and 318 for 15:01. The distributions for each dataset were plotted and normalized based on the number of peptide binders. We used the Aggrescan server [22] to calculate their aggregation propensities. The Aggrescan method is a bioinformatics strategy where experimental aggregation-propensity values per amino acid are used to calculate average values based on the input amino acid sequence. Specifically, an average value, using the four neighboring residues (*i.e*., two from the left and two from the right in the sequence), is calculated for each amino acid. Then, all the amino acid averages are summed, then normalized by the number of total residues in the input sequence and multiplied by 100. The final normalized value is used to compare sequences. We used as a reference value the Aggrescan score obtained for P10.

### 2.2 Design of P10 variants

#### 2.2.1 Modeling of P10 bound to a MHC class II allele

The starting complex to design P10 variants was the structure of MHC-II allele DRB1:01*01 with PDB id 1t5x. The bound peptide with sequence AAYSDQATPLLLSPR was used as a template to structurally model the P10 15-mer sequence QTLIAIHTLAIRYAN. We modelled the sequence by aligning 9-mer core regions of the peptides and mutating position-by-position using the package fixbb from Rosetta [23]. To predict the 9-mer core regions of both peptides, we implemented the NetMHCIIpan-4.0 tool [4]. The side chains of the complex were relaxed using Rosetta with the protein backbone fixed. Then, the refined protein-peptide structure was subjected to 200 nanoseconds (ns) molecular dynamics (MD) simulation with previous minimization and NVT/NPT equilibration, using GROMACS version 5.1 [24]. Two replicas with different random starting velocities were performed.

To run the simulations, the Amber99SB-ILDN protein force-field [25] was chosen, given previously validated results using the MHC-II and other protein-peptide systems as reference [21]. A TIP3P water model [26], a modified Berendsen thermostat [27], and a Parrinello-Rahman barostat [28] were used during the equilibration and production phases. The protein-peptide complex was solvated in a cubic box of water with periodic boundaries at a distance of at least 8 Å from any atom of the protein. Counterions of *Na*^+^ and *Cl*^−^ were included in the solvent to make the box neutral. The electrostatic interactions were calculated using the Particle Mesh Ewald (PME) method, with 1.0 nm short-range electrostatic and van der Waals cutoffs [29]. The equations of motion were solved with the leap-frog integrator [30], using a timestep of 2 femtoseconds (fs). A temperature of 310 K was chosen to perform the simulations. A summary of the parameters is available in Table 1.

**Table 1:** List of parameters and configurations used for the MD simulations.

**Parameter/configurations**	**Value/details**
Temperature	310 K
Force field	AMBER99SB-ILDN [25]
Solvation	TIP3P water model [26]. Cubic box with periodic boundaries of at least 8 Å and counterions
Electrostatic interactions	Particle Mesh Ewald with 1.0 nm cutoff [29]
Integrator	Leap-frog with timestep of 2 fs [30]
Minimization	Gradient-descent (1000 steps)
Equilibration (NVT)	100 ps using modified Berendsen thermostat [27]
Equilibration (NPT)	100 ps using Parrinello-Rahman barostat [28]
Production	200 ns (initial simulation). 100 ns (post-design simulations).

#### 2.2.2 Verification of β-strand region at the N-terminal region of P10

To check the formation of the β-strand in the peptide, we modelled P10 bound to allele DRB1*03:01 using the crystal structure with PDB id 1a6a, and bound sequence PVSKMRMATPLLMQA as template for the structural modelling of P10. The modelling and simulations were performed following the same steps described in the previous section. In addition, we mutated in the DRB1*01:01 crystal structure (PDB id 1t5x) only the TLIA fragment in the same aligned region and ran the MD simulation using the described parameters. Observables such as the peptide secondary structure and the generation of interactions with MHC-II chains were annotated.

To analyse the role of the β-strand in the MHC/peptide binding process, we extracted a set of 23 crystal structures from the PDB of various MHC-II alleles bound to peptides. The peptides were subjected to the DSSP software in order to annotate their secondary structure when bound to the MHC-II. This allowed us to verify how common is the formation of the encountered β-strand.

#### 2.2.3 Design protocol

To design the P10 variants, we used the PARCE protocol [21] that employs a stochastic search over the sequence of the peptide to efficiently explore the sequence space. At each step, the protocol selects a position in the peptide chain and an amino acid to mutate to. After the point mutation is generated, the system is subjected to all-atom MD simulations in explicit solvent to sample the conformations of the mutated peptide. After performing the mutation, a first minimization of the predicted side chain alone is performed. In order to relocate overlapping atoms and avoid clashes, a second minimization is run with the new amino acid and the water molecules surrounding it within 2 Å. Finally, a minimization of the full system is performed with a subsequent NVT equilibration of 100 picoseconds (ps). Then, the new system is sampled for 5 ns using the MD setup explained in Section 2.2.1, and a new mutation is performed following the same rules.

The acceptance of the mutation is based on a consensus by vote of six scoring functions evaluated over the structures from the MD simulations [31], which were Pisa [32], Firedock [33], BACH [34], [35], [36], ZRANK [37], IRAD [38] and BMF-BLUUES [39], [40]. If a particular number *n* of scoring functions agrees with negative scoring differences between peptide *A* (original) and peptide *B* (mutated), then the final consensus will accept the change between the peptides. Based on previous studies, a threshold of three (from six scoring functions), was defined to accept or reject the mutations. The starting structure of the protocol was the last frame of the P10 bound to the MHC-II allele DRB1:01*01 simulation (described above). More details of the protocol’s main steps are explained in the Supplementary Notes 1 and 2.

We defined two modification strategies and two sets of positions on the peptide to be modified. The first set of positions involved modifying only the TLIA fragment of P10, responsible for the β-strand formation found with the MD. The second included the same fragment (TLIA) plus the peptide flanking regions of P10 based on the 9-mer core prediction (see Section 2.2.1), which were found useful for multiple-allele MHC II peptide engineering [31]. After selecting the two sets of positions, we performed two different design runs defined according to the following filters for each:

*Design run 1:* New amino acids are selected randomly at the selected two sets of positions without filters. The methodology aims to explore the sequence space without bias.

*Design run 2:* Selection of amino acids able to decrease the potential peptide hydrophobicity, as well as increase the chances of being synthesized and solubilized during experimental phases. To fulfill the conditions, three bioinformatics filters were applied. Two consisted of empirical rules to account for solubility and synthesis issues associated with peptides. The rules describe violations raised by certain patterns or amino acid types found in the peptide sequence (see Supplementary Note 3 for details) [41]. The larger the number of violations, the lower the possibilities to validate the peptides experimentally. The third filter was the calculation of a peptide hydrophobic score using the Eisenberg hydrophobicity scale defined for proteinogenic amino acids [42]. In this strategy, we perform a uniform random mutation at the predefined sets of positions. The mutation is selected if the new sequence maintains the hydrophobic score lower than 3, the number of violations to the synthesis rules lower than 5, and the number of violations to the solubility rules lower than 2. The thresholds were selected after applying the three metrics in a group of known peptide binders of the MHC-II allele DRB1:01*01 [43].

### 2.3 Selection of P10 variants for experimental testing

In the PARCE protocol single mutations are accepted following a consensus-based approach with a set of scoring functions (see above, Supplementary Note 1 and 2). After finalizing the design runs, we used the scores to obtain an average rank per peptide per each design strategy. Specifically, all the accepted peptides were ranked using each scoring function, and the average rank over the six functions was calculated. Then, based on the average rank, and hydrophobicity and Aggrescan scores lower than the P10 reference (see Supplementary Figure 3), we prioritized 18 sequences with potentially better affinities and better properties than P10.

The prioritized peptides were subjected to additional MD simulations of 100 ns using the same setup explained in Section 2.2.1. The last half of the trajectory (*i.e.,* the last 50 ns) was used to calculate the average score using the same six scoring functions. With the final averages, a new average rank was calculated and used to re-rank the candidates for subsequent experimental validations. For the experiments, we also included the P10 original sequence, and the 13-mer Influenza peptide (PKYVKQNTLKLAT) as a control, which has been demonstrated is a good binder against the DRB1*01:01 allele [44]. The control peptide bound to MHC-II was also subjected to 100 ns MD simulation using the same crystal structure (PDB id 1t5x) to model the complex.

### 2.4 *In silico* aggregation analysis

The intrinsic aggregation and amyloid formation propensities were evaluated with the Aggrescan server employing default settings [22]. The Cordax algorithm [45] (https://cordax.switchlab.org/), a structure-based machine learning algorithm that models hexapeptides into a β-sheet fibril cores, was used to generate models of the amyloid core as steric zippers for a subset of ten P10 variants. Cordax uses complementary computational alternatives to determine the structural layout of putative amyloid fibril-forming segments based on 1402 hexapeptide sequences available in its library. Only the peptide/protein sequence is necessary to use this algorithm, and the server does not allow changes in any parameter. The output PDB files were visualized and edited with PyMOL.

### 2.5 Experiments

#### 2.5.1 Rate binding assays

The selected peptides were subjected to a gold-standard rate-binding assay against the MHC-II allele of reference. The Proimmune REVEAL® binding assay was used for that purpose, which uses antibody-labelled peptides that emit a signal if native conformations of the complexes are detected. Based on a control provided by Proimmune, we obtained per peptide a score (between 0 and 100) that measures a proxy affinity toward the MHC-II allele with two data points, one at 0 h and a second after 24 h. Based on these two measures a stability index is assigned. The peptides were synthesized using the Prospector PEPscreen® technology with high purity standards based on quality controls obtained by MALDI-TOF mass spectrometry [46].

#### 2.5.2 *In vitro* aggregation assays

For *in-vitro* aggregation assays, P10 and a total of ten selected P10 variants were purchased from Genscript with purity above 95 % and diluted in DMSO (Sigma-Aldrich code D8418) 100 % to a final concentration of 5 mM (stock solution) and kept at −20ºC for storage.

P10 and its variants stock solutions were diluted in PBS at 20 μM; pH 7.4 at 37ºC for 18 h under agitation. To measure light scattering (LS), samples were excited at 320 nm while emission was collected at 320 nm in the spectrofluorometer Jasco FP8200 (Jasco Corp., Tokyo, Japan). Aliquots of the aggregation suspension were diluted in the presence of Thioflavin-T (Sigma-Aldrich code T3516) (ThT; 50 μM and peptide 20 μM), a specific fluorescent probe for AF. ThT fluorescence emission was measured at 485 nm by exciting the samples at 450 nm. Congo red (Sigma-Aldrich code C6767) binding assays, another amyloid specific probe, were performed according to Palhano [47]. The samples (50 μL) were centrifuged at 17,000 X g for 30 min, and the pellet was incubated with 10 µM Congo red solution for 5 min. The absorbance was measured at 540 and 477 nm and the fraction of bound Congo red was determined by the following formula [mol of bound Congo red/mol of protein = OD_540_/25,295 – OD_477_/46,306].

#### 2.5.3 Transmission electron microscopy (TEM)

5 mL of each peptide suspension (100 µM) was absorbed onto 200-mesh carbon-coated copper grids (Pelco, Ref: 01800 F) for 5 min and then blotted to remove excess material. Negative staining was performed by adding 5 mL of 2 % (w/v) uranyl acetate. Samples were dried on air for 3 min. The grids were imaged with a Jeol 1200 electron microscope (Jeol Ltd.) operating at a 60 kV acceleration voltage.

## 3 Results

### 3.1 High-affinity MHC-II epitopes are more disposed to aggregate *in silico*

**Fig. 1:**
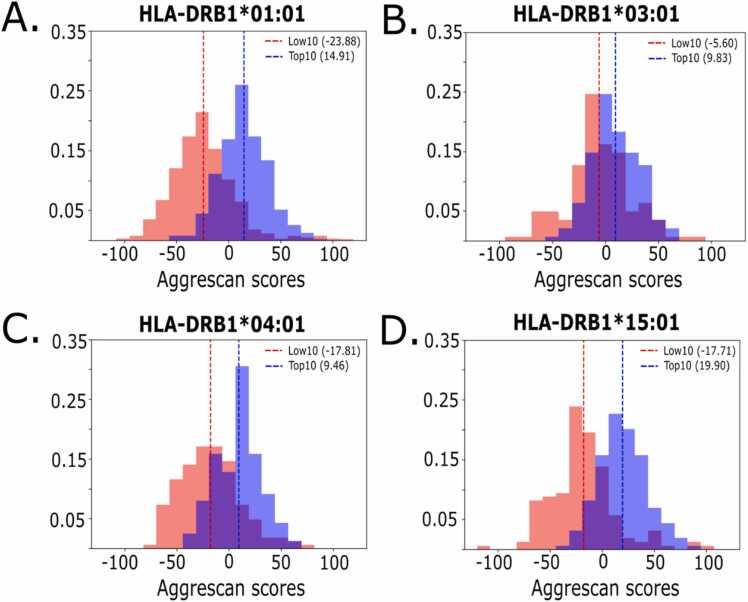
Aggregation-propensity distributions for high and low binders MHC-II epitope sets. Aggrescan scores were used as an aggregation propensity measure for the higher (Top10; blue) and lower (Low10; red) affinity binders to MHC-II alleles (A) DRB1*01:01, (B) DRB1*03:01, (C) DRB1*04:01 and (D) DRB1*15:01. The frequency values were normalized based on the number of peptide binders per dataset. The Aggregation Propensity Average (AggP) values are displayed in parenthesis in each panel.

To characterize the connection between the aggregational profile and affinity of different MHC-II epitopes, we used sequences from peptides available on the IEDB database that have an experimentally measured binding affinity to MHC-II. We assessed their aggregation propensity using Aggrescan, which predicts an aggregation propensity score using the primary sequence [22]. The higher the Aggrescan value the higher the tendency to aggregate (*i.e*., Amyloid-β 1–42 peptide = 6.4). As seen in Fig. 1, the top 10 % (Top10; blue) of the highly-binding epitopes (ranked with the experimental data) have higher Aggrescan scores when compared with the 10 % worst (Low10; red) epitopes. The Low10 class has predominantly negative scores for all alleles. For example, the Top10 binders to DRB1*01:01 shows an Aggregation Propensity Average (AggP) of 14.91, while Low10 epitopes show AggP of −23.88 (Fig. 1A). The same is shown for the other alleles (see the other AggP values in each panel).

These results indicate a potential correlation between aggregation propensity (as determined by Aggrescan) and MHC-II epitopes binding, which were found for different HLA alleles. To validate these *in silico* results regarding MHC-II affinities and aggregation propensities, we selected the P10 peptide as a template for designing variants with different binding affinities and aggregation propensities to search connections.

### 3.2 Modelling of P10 and MD simulations

**Fig. 2:**
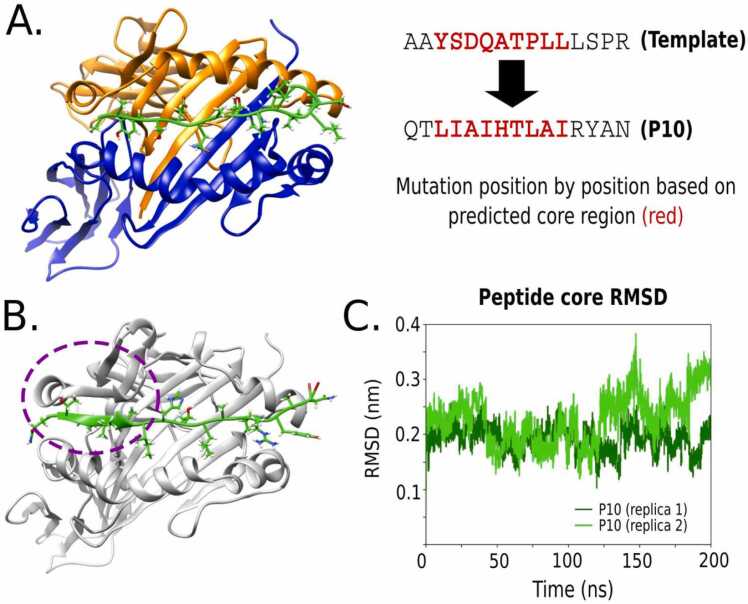
Summary of the modelling and MD simulation of P10 bound to the MHC-II allele. (A) Crystal structure of the complex template (PDB id 1t5x), and the mutation strategy to generate the bound P10 sequence by replacing position-by-position the amino acids from the peptide template. (B) Formation of the β-sheet fragment between P10 and MHC-II after 200 ns MD simulation (highlighted by a circle). (C) C-alpha RMSD of the peptide core from the initial structure for two MD replicas.

To design variants of P10 that vary in their affinity to MHC-II and aggregation propensities, we used the PARCE protocol to modify specific positions on the sequence bound to the MHC-II allele DRB1*01:01 (see Methods). A key aspect previous to designing the variants was to perform a large conformational sampling of the initial complex MHC-II/P10 complex using MD and looking for convergence of the peptide interactions. For that purpose, we started with a PDB template of the MHC-II bound to a 15-mer peptide (Fig. 2A). The predicted core region of the peptide template aligns at the same position of P10 core, which facilitated the replacement point-by-point of the new amino acids. Interestingly, after running 200 ns of MD simulations, we observed the formation of a short β-strand in the ^2^TLIA^6^ fragment of P10 with a segment of the MHC-II α-chain, namely the segment ^53^SFAE^56^ (circle in Fig. 2B). This region interacts with the peptide β-strand fragment in the cleft that undergoes a α-helix to β-strand transition. For the two independent MD replicas, this β-strand is formed and the RMSD of the peptide core region remains stable (Fig. 2C).

Given the behaviour of P10 using the DRB1*01:01 allele, we decided to verify if the β-sheet would be generated by modelling P10 with a different allele, in this case DRB1*03:01 (PDB id 1a6a). After 200 ns of simulation, we observed the formation of the same β-sheet in the ^2^TLIA^6^ fragment of P10 in complex with this new allele (see Supplementary Figure 1A). We speculate that this P10 fragment could be responsible for inducing this β-sheet formation, independent of the remaining amino acids in the peptide sequence. To validate the latest, we inserted the TLIA fragment onto the original 15-mer template generating the sequence AA**TLIA**ATPLLLSPR. After running the 200 ns simulation with DRB1*01:01, we observed again the formation of the same β-sheet (see Supplementary Figure 1B), which hints to the role of this region not only for binding, but also for the potential aggregation of P10, since β-strands are enrolled in amyloid fibril formation as was observed with P10 in solution [20].

To complement the analysis, we searched for PDB crystal structures of multiple MHC-II alleles bound to peptides of different nature. After a detailed revision of the peptides secondary structure, most of them did not form the β-sheet at the N-terminal region, except for one peptide from influenza virus (PGMMMGMFNMLSTVLGVSIL; PDB id 6qza) that has a very high Aggrescan score (51) and, in principle, tends to form aggregates (see Supplementary Table 1).

### 3.3 Design of P10 variants

Based on these findings, we decided to generate peptide variants by modifying the TLIA region and flanking amino acids of P10 to evaluate whether aggregation propensity and affinity to MHC-II would correlate.

#### 3.3.1 Design run 1

**Fig. 3:**
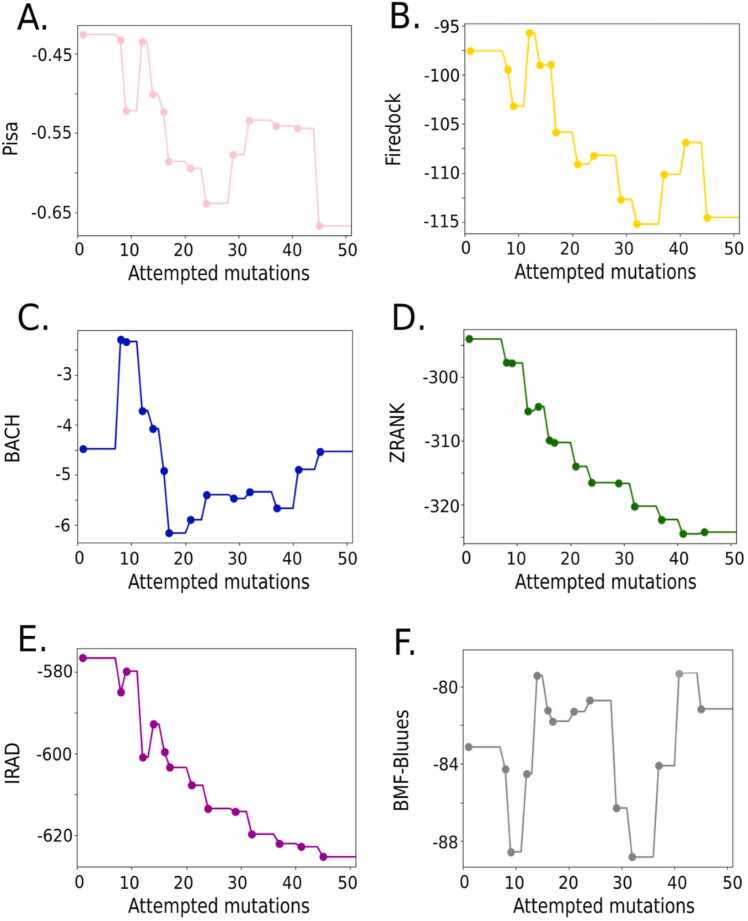
PARCE score evolution for the design run 1 using the TLIA fragment of P10 associated with the formation of the β-strand. A total of 50 mutation attempts were tried using the six scoring functions: (A) Pisa [32], (B) Firedock [33], (C) BACH [34], [35], (D) ZRANK [37], (E) IRAD [38] and (F) BMF-BLUUES [39], [40].

The initial design run involved random mutations using two sets of positions: the TLIA region alone and TLIA together with flanking amino acids of P10. For the first set, we attempted 50 mutations, and for the second, 100 mutations, with an acceptance ratio in both cases around 20–30 %. The accepted sequences, sorted based on the average ranks using the six scoring functions, are available for the TLIA region, and for both TLIA and flanking regions position sets in Supplementary Table 2 and 3, respectively. The evolution of the six scoring functions mutating only the TLIA region is shown in Fig. 3, and for the TLIA plus flanking amino acids is shown in Supplementary Figure 2.

As shown in Fig. 3, the scoring functions are, on average, minimizing their value through the trial of multiple mutations. Nonetheless, due to the nature of the stochastic search and the consensus criteria, the scores can also increase to overcome local barriers. In the case of modifying TLIA and the flanking amino acids, we found that many accepted sequences had many hydrophobic and charged amino acids, which can affect the synthesis and evaluation of their activity, as well as the probabilities to aggregate (see Supplementary Table 2 and 3). To solve that, we included additional filters to guide the mutation strategies for improving their physico-chemical properties.

#### 3.3.2 Design run 2

For this strategy, we used the same two sets of peptide positions, but we filtered the mutated sequences using some amino acid properties, including hydrophobicity scores and two empirical rules to infer potential violations to synthesize and solubility (see Methods Section 2.2.2). We obtained a similar 20–30 % acceptance ratio, and the accepted sequences were sorted based on the average rankings (Supplementary Tables 4 and 5). In this scenario, we obtained less hydrophobic sequences that can have higher chances of success in synthesis during the experiments. In addition, we compare the hydrophobicity values for each peptide with the aggregation scores predicted with Aggrescan, finding a correlation between the two (see Supplementary Figure 3). This allowed us to filter less hydrophobic and potentially less prone to aggregation P10 variants for further experimental phases.

#### 3.3.3 Final ranking

From both design runs, and after calculating the average ranks, we found 46 sequences with potential better affinities for MHC-II than the reference peptide P10. From there, we selected 18 sequences with lower hydrophobicity scores than P10, which potentially have lower chances to aggregate (see Supplementary Figure 3). The prioritized 18 sequences in complex with the MHC-II receptor were subjected to longer MD simulations of 100 ns, and a similar average rank was calculated. The final set of peptides with their corresponding Aggrescan scores, hydrophobicity values and violation of empirical rules are shown in Table 2.

**Table 2:** List of final peptides selected after running 100 ns MD simulations and calculating the average rank. The peptide sequence, Aggrescan score prediction, hydrophobicity value and number of violations to empirical solubility and synthesis rules are included. The P10 sequence is in bold. The N-terminal region that is different among all the peptides are underlined.

**Peptide**	**Sequence**	**Aggrescan**	**Hydro**	**Sol. rules**	**Syn. rules**
V1	QTLLDIHTLAIRYAN	12.7	1.88	1	1
V2	QNLHAIHTLAIRYAN	-0.7	1.21	2	1
V3	QPFPDIHTLAIRYAN	0.6	1.24	2	1
V4	QPFCDIHTLAIRYAN	8.1	1.41	1	2
V5	QPFQDIHTLAIRYAN	-6.6	0.27	1	1
V6	QTLTAIHTLAIRYAN	12.4	2.29	1	3
V7	QGLPAIHTLAIRYAN	9	2.99	2	3
V8	QMHHAIHTLAIRYAN	-5	1.17	2	2
V9	QPFVDIHTLAIRYAN	16	2.2	1	1
V10	QGLKAIHTLAIRYAN	4.2	1.37	2	1
V11	QTLQAIHTLAIRLAN	5.3	2.29	2	3
V12	QSHHAIHTLAIRYAN	-11.4	0.35	2	1
V13	QLLHAIHTLAIRYAN	13.6	3.05	2	1
V14	QTYHAIHTLAIRYAN	3.9	1.14	1	1
V15	QNHHAIHTLAIRYAN	-16.8	-0.25	2	1
V16	QMLHAIHTLAIRYAN	11.1	2.63	2	2
V17	QTTISIHTLAIRYAN	16.3	1.81	1	3
V18	MTGDHIHTLAIRYFN	-2.3	1.9	1	1
**P10**	**QTLIAIHTLAIRYAN**	**28.3**	**3.72**	**2**	**3**

For all the peptides, we verified the final bound conformations after running the long MD simulations. We found that all of them miss the β-region at the N-terminal part suggesting that the strong binding potential of P10 to MHC-II might be disrupted. For five of the selected peptides, we show the bound conformation in Supplementary Figure 4. We also show for the 18 peptides, curves of the RMSD during the 100 ns simulations (Supplementary Figures 5, 6 and 7) in order to assess their conformational stabilities. None of the peptides reported RMSD values over 5 Å, and no significant conformational changes of the binding poses were observed.

### 3.4 Experimental rate binding assays

**Fig. 4:**
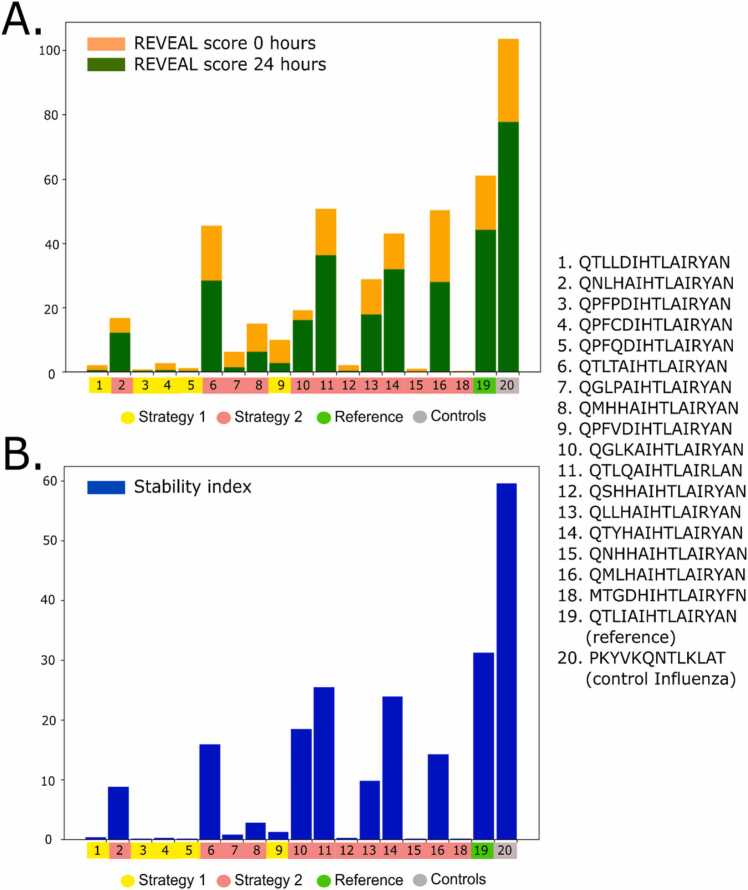
Experimental REVEAL binding score at 0 h (orange) and at 24 h (green) (A) and stability index (B) for the selected peptides (# 1–18), the reference P10 sequence (#19) and the Influenza control (#20). The variants are assigned to the number given in Table 2. Each peptide in the x-axis is represented by the design strategies explained in Methods 2.2.3.

The peptide candidates, the P10 sequence and an Influenza peptide control were synthesized and analyzed using the ProImmune REVEAL® MHC-peptide binding assay to determine their level of incorporation into the MHC-II allele DRB1*01:01 (see Methods). Binding to MHC molecules was compared to an additional T-cell epitope used as a positive control peptide with strong binding properties. The experimental values indicating the proportion of assembled complexes that have remained after the 24 h incubation is available in Fig. 4.

Five of the designed P10 variants (V6, V11, V13, V14 and V16) remain with a similar activity to P10 at 0 h despite the modifications that disrupt the β-strand (Fig. 4A). On the other hand, if we focus on the stability index, a similar subgroup has high stability (Fig. 4B). Interestingly, there is a diverse range of binding and stability results. We selected a list of ten peptides that were divided into two subfamilies (SF) based on the experimental affinity scores: SF-high includes the peptides with high affinity for MHC-II (P10, V6, V10, V11, V14 and V16), and SF-low, which includes the peptides with low affinity for MHC-II (V1, V3, V5, V7 and V15). These were prioritized for the aggregation analysis.

### 3.5 *In silico* model of steric zippers

AF are mainly formed by the cross-β fold in which the core forms an interdigitated structure, named steric zippers [48] because the lateral chains of the amino acids from a β-strand intercalated with the lateral chains from the amino acids from the β-strand in front of it creating a tight, dry interface. Interestingly, these zippers have been observed in AF extracted from patients with amyloidosis [49].

**Fig. 5:**
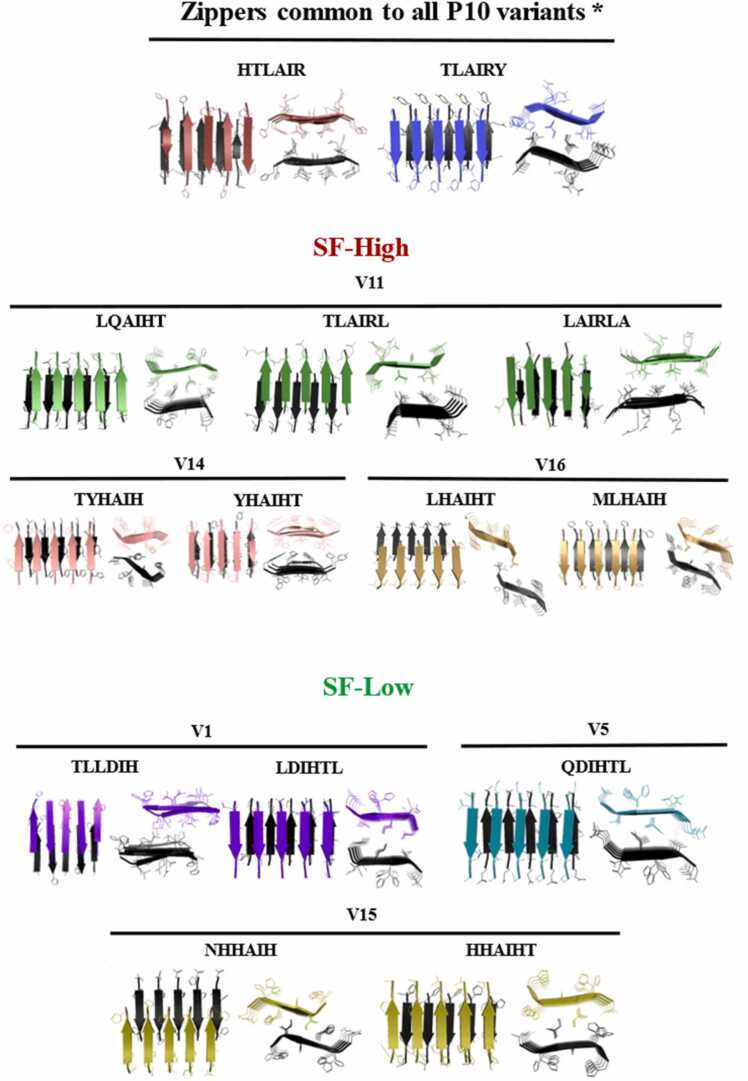
Cordax modeling of steric zippers predicted to form by P10 variants. (A) Zippers common to all sequences except V11 (*). (B) Steric zippers predicted in SF-high composed of peptides V11, V14 and V16 (V6 and V10 are not shown because they have only two predicted consensus zippers). (C) Steric zippers predicted in SF-low composed of peptides V1, V5 and V15 (V3 and V7 are not shown because they have only two predicted consensus zippers).

Since P10 aggregates into AF *in vitro*, we investigated whether the peptides probed with ProImmune REVEAL® binding assays were able to form amyloid aggregates as well. To address this question, we initially submitted the ten selected sequences of the P10 variants (V1, V3, V5, V6, V7, V10, V11, V14, V15 and V16) to Cordax, an amyloid structure predictor able to model a given structure into steric zippers common to amyloids. For most of these P10 variants, regardless of their SF, at least two types of consensus steric zippers encompassing the sequence ^7^(H)TLAIR(Y)^13^ were predicted. The exception was V11, which has a leucine replacing the tyrosine 13, which leads to the formation of ^8^TLAIRL^13^, ^9^LAIRLA^14^, and ^3^LQAIHT^8^ zippers (Fig. 5A and B). Besides the two zipper types observed in all peptides (henceforth consensus zippers), V14 and V16 from SF-high can form zippers in the regions ^2^TYHAIH^7^/ ^3^YHAIHT^8^ and ^2^MLHAIH^7^/^3^LHAIHT^8^, respectively. Regarding the SF-low, in V1, V5 and V15 additional zippers were observed (Fig. 5A): V1: ^2^TLLDIH^7^ and ^4^LDIHTL^9^; V5: ^4^QDIHTL^9^ and V15: ^2^NHHAIH^7^ and ^3^HHAIHT^8^ (Fig. 5B). It is important to highlight that V3 and V7 from SF-low and V6 and V10 from SF-high are not depicted in Fig. 5 because they only form the two consensus zippers ^7^HTLAIR^12^ and ^8^TLAIRY^13^. Regarding the P10 zippers structure, it was previously shown that its regions ^2^TLIAIH^7^, ^3^LIAIHT^8^, ^4^IAIHTL^9^, ^7^HTLAIR^12^ and ^8^TLAIRY^13^ are able to interact and form steric zippers as well [20].

### 3.6 *In vitro* aggregation experiments with P10 variants

**Fig. 6:**
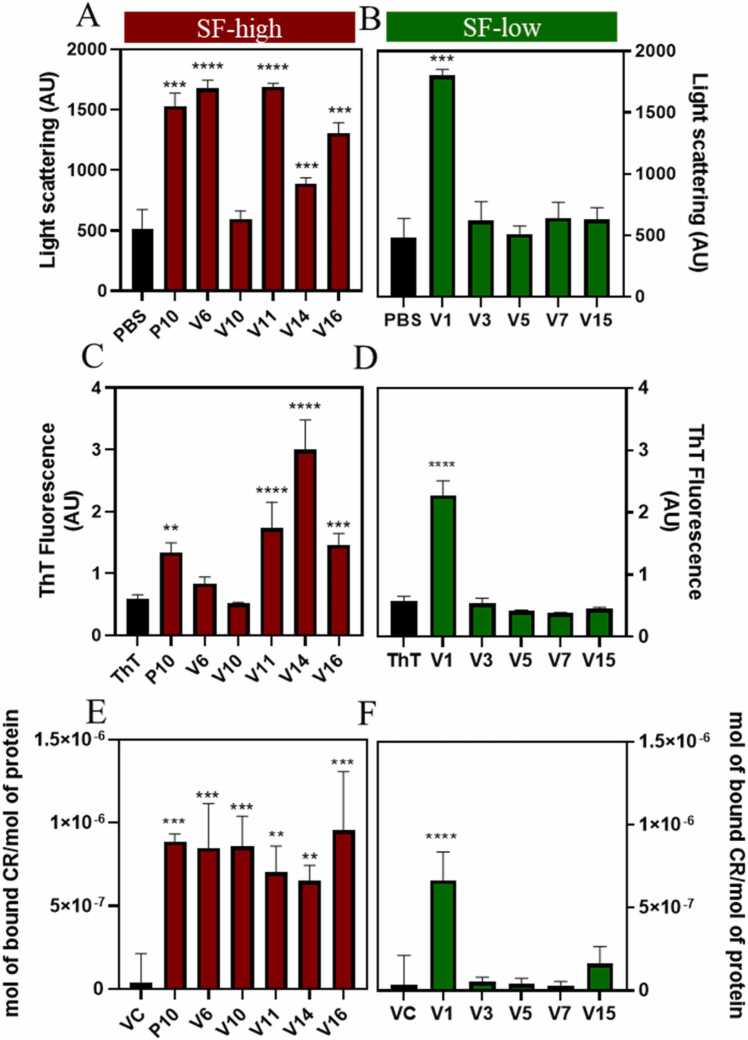
*In vitro* aggregation profile of the ten peptides derived from P10 belonging to SF-high (high affinity for MHC-II; red) and SF-low (low affinity for MHC-II; green). 20 μM of each peptide was incubated in PBS, 37 ^o^C for 18 h and Light Scattering (A and B), Thioflavin-T (ThT; C and D) and Congo red (CR; E and F) binding were measured. Data were analysed by one-way ANOVA. Significant difference relative to control group: **** p < 0,0001, *** p < 0,0009, ** p < 0,0025 and * p < 0,05.

Cordax modeling has shown that the P10 variants can, in principle, form steric zippers, in some cases in more than two sequence regions, suggesting that these peptides could form amyloid fibrils in solution. Regarding Aggrescan analyses, peptides from SF-high presented higher aggregation propensity score (AggP = 7.38), when compared with SF-low peptides (AggP = −0.22; Aggrescan score for P10 = 28.3; Table 2). The ten peptides and P10 were synthesized for aggregation studies. Peptides were diluted at 20 μM; pH 7.4 at 37ºC for 18 h. Afterward, light scattering (LS), Thioflavin T (ThT) and Congo red (CR) binding were evaluated. These two probes are specific for amyloid fibrils. As seen in Fig. 6A, with the exception of V10, the peptides from the SF-high presented high values of LS comparable to P10 suggesting the formation of aggregates. Regarding ThT binding, in some cases the peptides bound even more ThT than P10, except for V6 and V10 that did not bind this amyloid-specific probe. However, all peptides from this SF-high group bind CR, including V6 and V10. Altogether, these data suggest that the peptides that displayed higher affinities for MHC-II (SF-high) underwent aggregation when in solution. Curiously, the peptides from SF-low did not present an increase in LS, ThT or CR binding, when diluted in aqueous solution, except for V1, which behaved like P10. Thus, except for V1, the peptides that displayed lower affinities from MHC-II (SF-low) seem to be soluble under these conditions.

**Fig. 7:**
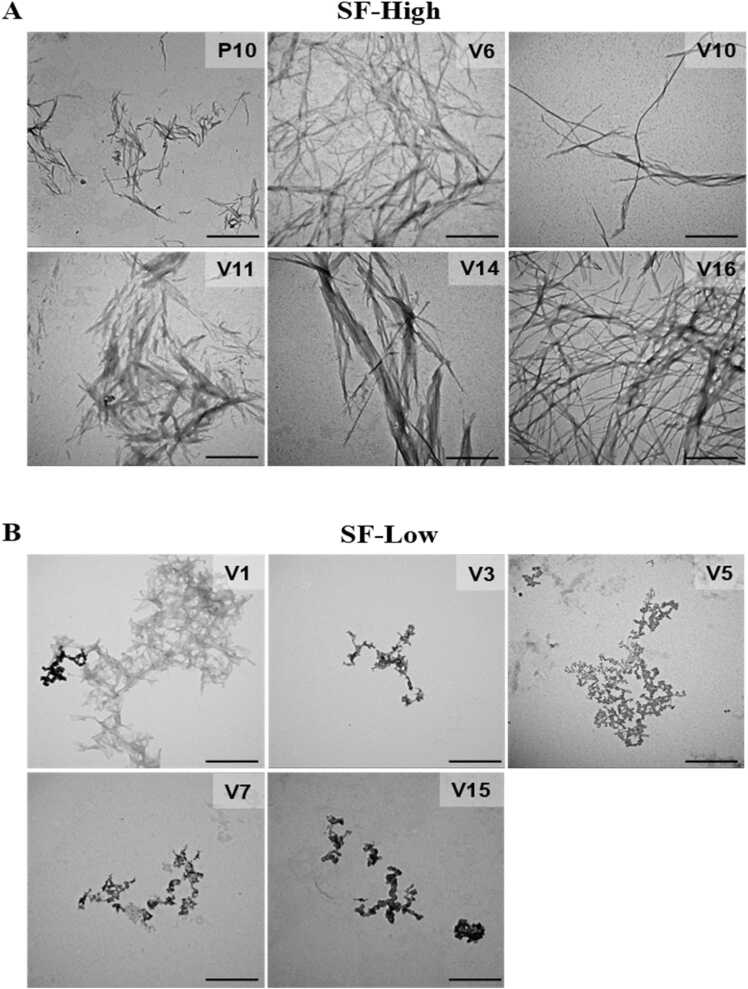
Peptides derived from P10 presenting high affinities for MHC-II (SF-high) form amyloid fibrils in solution. TEM images of SF-High (A) and SF-Low (B). Each peptide was incubated at 20 μM in PBS for 18 h at 37 ^o^C. Size bar: 500 nm.

TEM imaging was performed to gain insights into the architecture of the aggregates formed in solution (Fig. 7). As seen, all peptides from SF-high formed mature amyloid fibrils, even V6 and V10, which did not bind ThT but bound Congo red. It is possible to see some differences in fibril morphology; some of them, like those from V14, have a ribbon-like appearance. Interestingly, the peptides from SF-low, when diluted in solution, did not form mature amyloid fibrils, but only amorphous aggregates. It must be emphasized that we had to search TEM grids exhaustively in order to find these amorphous structures. In accordance with Aggrescan scores and tinctorial experiments, V1 is an exception and amyloid fibrils were observed in the images (Fig. 7B).

## 4 Discussion

A motivation of this work was to assess if P10’s aggregation behaviour could have a relevance to its great antigenic potential *via* the binding to MHC-II [20]. This prompted us to explore deeper whether there is a connection between aggregation propensity of a given peptide and its binding affinity and stability to MHC-II. First, we studied a set of MHC-II epitopes from the IEDB database split into high and low binders with different ranges of aggregation propensities. We observed statistically significant differences between the two sets in terms of the aggregation propensities and the affinities to multiple MHC-II alleles, prompting us to use P10 as a benchmark for further detailed analysis.

P10 presents in its sequence the main epitope domain HTLAIR that interacts with MHC-II and induces a protective immune response [50]. After running MD simulations of P10 bound to MHC-II, we found that the segment ^53^SFAE^56^ from the MHC-II α1 subunit underwent a marked conformational change by assuming a small β strand. This strand forms a β-sheet with the segment of P10 ^2^TLIA^6^. This conformational change is likely related to this specific fragment of P10, given a similar adoption of a β-strand after it was inserted into a different peptide that was originally bound in an extended conformation. Searching in several structures of MHC-II bound to different peptides in the PDB, we found only the case of a CD4+ T cell epitope named GMF (PGMMM**GMF**NMLSTVLGVSIL) from the polymerase basic-1 protein (PB-1) of the influenza virus [51] to adopt a similar β-sheet conformation. When GMF binds into the cleft of MHC-II, the segment ^6^GMF^8^ adopts a β-strand, which forms a β-sheet with the residues SFA from the α-subunit of MHC-II. The question that remains open is whether this β-strand transition (induced by these peptides upon binding) is a particular case, or a plausible determinant for a stronger affinity of a given peptide to the MHC-II. Interestingly, the Aggrescan score of GMF is 51, a value even higher than that of P10 (28.3).

To study more in detail the relation between aggregation and MHC-II binding, we designed 18 peptides from P10 using PARCE [21]. Their experimental affinity and stability to MHC-II allele DRB1*01:01 was probed using the Proimmune REVEAL® binding assays. We split the peptides into two subfamilies (SF): the one that presented low - or no- stability/affinity (SF-low), and the other SF had high stability/affinity toward the MHC-II allele (SF-high). Most of the peptides from the SF-low presented negative Aggrescan scores (exceptions are V1, V4, V7 and V9 out of 10 peptides) while the Aggrescan scores for the peptides from SF-high were positive (exception is V2 out of 7 peptides). This initial analysis corroborates what we observed with the massive analysis of the IEDB database, where we noticed a positive correlation between aggregation propensity and affinity for MHC-II.

By experimentally probing the aggregation, we found that all members of the SF-high, including P10, bound ThT, Congo red or both, and presented, as seen by TEM, a fibrillar morphology confirming they form amyloid fibril when in solution. We do not have an explanation of why V6 and V10 did not bind ThT, a property common to most amyloid fibrils [52]. It is possible that the AF formed by these two peptides are unable to accommodate the benzylamine and benzathiole rings of ThT in their grooves, which gives this probe a high quantum fluorescence yield. V6 and V10 are the only two peptides from the SF-high that are predicted to form only the two consensus zippers. More structural information would be necessary to understand why these fibrils do not bind ThT. Except for V1, all members of the SF-low did not bind ThT or Congo red and did not show the presence of amyloid fibrils by TEM. Only amorphous aggregates were observed but they seem to represent a minor fraction of the population in solution. V1, which has an Aggrescan score of 12.7, is the only peptide from SF-low that kept some sequence similarities with P10 (P10 → **QTL**IA; V1 → **QTL**LD). This could explain why V1 kept some of its aggregation propensity.

Overall, there are several factors that determine whether a peptide is displayed or not in MHC-II, including its uptake route, accessibility of the native antigenic protein to proteases, concentration, as well as structural properties such as size, primary sequence, and complementarity to the MHC-II pockets. Here, we describe an additional layer of information that could be used to predict good and poor epitope-binders to MHC-II, which is its aggregation propensity. According to the Aggregation hypothesis of antigen selection as enunciated by Forsdyke [53], [54], homoaggregation of antigenic peptides, predominantly an entropy-driven process favoured by an increase in temperature (pyrexia), would colocalize identical peptides, thus facilitating their collective presentation. As shown in our previous study of P10 [20], its aggregation into amyloid fibrils was more prominent at neutral pH than at acidic pH. There are studies proposing that aggregation of antigens might be a strategy of phagocytic cells to concentrate and preserve the integrity of these antigenic peptides before their insertion into the MHC-II cleft and displacement of CLIP from the cleft. Interestingly, the core sequence of CLIP is a nine-residue fragment (MRMATPLLM) with an Aggrescan score of 20.1, suggesting it has an aggregation propensity similar to the high affinity peptides here reported. The affinity of CLIP for MHC-II can vary largely and this variation could be associated with the ability of a peptide to displace it from the cleft. When released from the MHC-II cleft, it would be possible that CLIP undergoes aggregation for storage. In principle, it is counterintuitive why a peptide with a high aggregation propensity would present a higher affinity for MHC-II cleft, but in the light of the "Aggregation Hypothesis" for antigen selection it might have physiological significance.

In the last years, progress has been made in the development of bioinformatics tools to estimate and predict binding affinities between MHC-II alleles and antigenic peptides, although false positives remain. It is possible that the lack of an accurate algorithm has to do with the MHC-II’s fluctuations/dynamics that influence epitope recognition and stable binding. Most of the X-ray structures so far resolved only capture the ground state of the MHC-II and they present structural similarity. Complementary techniques such as MD simulation, H-D exchange and NMR among others are necessary to build the overall picture of the intimacy of MHC-II in the free and bound states. This task scales in complexity when we envision a large number of MHC-II alleles and the repertoire of possible epitopes to bind them. Moreover, there are other partners that stabilize pMHC-II such as HLA-DM (DM), which influences peptide editing and biases a peptide-exchange reaction. DM is a peptide-exchange factor that removes CLIP from the cleft of MHC-II replacing it with an antigenic peptide to be displayed at the plasma membrane.

By using mass spectrometry in combination with plasmon resonance binding experiments and crystal structure determination, Painter and colleagues [55] described an intermediate state of HLA-DR1 in the region of the 3_10_ helix (α45–50) and the adjacent extended region (α51–54) of the α-subunit, a region that includes the structural modifications here observed. This study showed a prominent role of αF54, which displays important sensitivity to DM-mediated peptide release. For example, the variant of MHC-II αF54C resulted in a protein with greater susceptibility to DM-mediated peptide release, revealing the structural alterations that make MHC-II more receptive to DM. In an additional study, Painter and colleagues [56] mapped the conformational heterogeneity of 41 peptide-MHC-II complexes (pMHC-II) and their data showed again that the 3_10_-helical region (α45–54), the kink region in the β1-helix (β62–71) and the β2-domain (β105–112) were the most heterogeneous regions of the protein upon binding. Our MD simulation data pointed to a determinant role of the region of the α subunit of MHC-II nearby segment ^53^SFAE^56^ that is close to these previously characterized dynamic regions.

Because of the "Aggregation Hypothesis" and the pH effect, we envision that P10 and other antigenic peptides, when cleaved from their harbouring protein during antigen processing in early/late endosomes (pH > 5), might form aggregates very fast inside these compartments until the pH is acidified by the fusion with lysosomes, generating the endolysosomes (pH < 5). This brings about the dissociation of the peptides from the aggregates to bind into the MHC-II cleft, followed by migration to the cell membrane and presentation to another immune cell. It has been shown that several peptidic hormones are stored as AF in the cells being released promptly when necessary [57]. Together with other peptides and proteins that adopt an amyloid fold with biological function, they are called functional amyloids. The data presented suggest that antigenic peptides can aggregate into AF, at least *in vitro*, constituting a reservoir of optimal antigens to MHC-II leaving the soluble peptides more susceptible to proteolysis. This would represent another example of functional amyloids here described for the first time. How general this phenomenon is an object for further investigations.

## Funding

This work has been supported by MinCiencias, University of Antioquia, Colombia, the Max Planck Society, Germany and the Simons Foundation, United States. We acknowledge the Federal Brazilian Funding Agencies, Capes and 10.13039/501100003593CNPq, and the State Funding Agency of Rio de Janeiro, Faperj, for the financial support and fellowships.

## CRediT authorship contribution statement

**Rodrigo Ochoa:** Conceptualization, Methodology, Software, Writing. **Thyago R. Cardim-Pires:** Methodology, Investigation, Writing. **Ricardo Sant’Anna:** Methodology. **Pilar Cossio:** Conceptualization, Supervision, Project administration, Writing. **Debora Foguel:** Conceptualization, Supervision, Project administration, Writing.

## Declaration of Competing Interest

The authors declare that they have no known competing financial interests or personal relationships that could have appeared to influence the work reported in this paper.
