# Supplementary material for: Connection between MHC class II binding and aggregation propensity: The antigenic peptide 10 of Paracoccidioides brasiliensis as a benchmark study
Source: Comput Struct Biotechnol J. 2023 Feb 18;21:1746–58. doi: 10.1016/j.csbj.2023.02.031 (PMC9986244; doi:10.1016/j.csbj.2023.02.031)
Supplement: Supplementary file 1 — Supplementary material [file mmc1.docx]

**Supplementary Information**

**Connection between MHC class II binding and aggregation propensity: the antigenic peptide 10 (P10) of *Paracoccidioides brasiliensis* as a benchmark study**

Rodrigo Ochoa^1,2^, Thyago R. Cardim-Pires^3^, Ricardo Sant’Anna^3^, Pilar Cossio^1,4,5,*^, and Debora Foguel^3,*^

^1^Biophysics of Tropical Diseases, Max Planck Tandem Group, University of Antioquia, 050010 Medellín, Colombia.

^2^Medicinal Chemistry, Boehringer Ingelheim Pharma GmbH & Co KG, 88397 Biberach/Riss, Germany

^3^Instituto de Bioquímica Médica Leopoldo de Meis, Universidade Federal do Rio de Janeiro, Rio de Janeiro, Brazil

^4^Center for Computational Mathematics, Flatiron Institute, New York 10010, United States

^5^Center for Computational Biology, Flatiron Institute, New York 10010, United States

**Supplementary Tables**

**Table S1.** Crystal structures of MHC class II receptors bound to different peptides. The PDB id, peptide sequence, origin organism/antigen, Aggrescan score and secondary structure are reported. The secondary structure categories are B (β-bridge), H (ɑ-helix), E (β-strand), S (bend), T (turn) and G (3/10 helix). The only complex (PDB id 6qza) reporting the β-sheet fragment at the N-terminal region is in bold.

| **PDB id** | **Peptide** | **Secondary structure** | **Aggrescan** | **Origin** |
| --- | --- | --- | --- | --- |
| 1a6a | PVSKMRMATPLLMQA | --EE---------- | 10.5 | CLIP |
| 1aqd | VGSDWRFLRGYHQYA | --EE---------- | -15.3 | HLA-A2 |
| 1fv1 | NPVVHFFKNIVTPRTPPPSQ | -----B------------- | 5.5 | Myelin |
| 1fyt | PKYVKQNTLKLAT | -B---------- | -8.2 | Influenza |
| 1h15 | GGVYHFVKKHVHES | -EE---------- | 1 | Epstein Barr |
| 1klg | GELIGILNAAKVPAD | -EE----------- | 12.3 | Melanoma - Human |
| 1sje | PEVIPMFSALSEGATP | -B---------BTTB- | 12.7 | HIV |
| 1sjh | PEVIPMFSALSEG | -B---------- | 25.2 | HIV |
| 1t5x | AAYSDQATPLLLSPR | -B---------S-- | -5.4 | Yeast - Superantigen |
| 1zgl | VHFFKNIVTPRTPGG | --EE---------- | 7.2 | Myelin |
| 2fse | AGFKGQQGPKGEPG | ------------- | -59.5 | Collagen |
| 2q6w | AWRSDEALPLGS | -B--------- | -28.3 | Integrin - Auto |
| 2seb | AYMRADAAAGGA | -EE-------- | -31.1 | Collagen |
| 3c5j | QVIILNHPGQISA | -B---------- | 13.3 | Elongation factor |
| 3pgd | KMRMATPLLMQALPM | -B---------S-- | 16.4 | CLIP |
| 3qxa | PVSKMRMATPLLM | -TTB-------- | 12.3 | CLIP |
| 4aen | MKMRMATPLLMQALPM | -----------EE-- | 16.1 | CLIP |
| 4h1l | QHIRCNIPKRISA | -B---------- | -18.3 | Mimotope |
| 4h25 | QHIRCNIPKRIGPSKVATLVRR | -B--------S-S-------- | -4.8 | Mimotope |
| 4h26 | QWIRVNIPKRI | -B-------- | 2 | Mimotope |
| 4i5b | VVKQNCLKLATK | ----------- | -4.8 | Influenza |
| 4ov5 | GSDARFLRGYHLYA | --B---------- | -8.5 | HLA-A2 |
| **6qza** | **PGMMMGMFNMLSTVLGVSIL** | **----EEEE------------** | **51** | **Influenza** |

**Table S2.** List of ranked peptides based on the average scores that were predicted with the PARCE protocol using the first design run strategy based on only random mutations. The changes are made only in the TLIA fragment of P10 that forms the β-sheet after 50 mutation attempts. Aggrescan values are included. The P10 peptide is in bold.

| **Peptide** | **Aggrescan** |
| --- | --- |
| QGLKAIHTLAIRYAN | 4.2 |
| QTYHAIHTLAIRYAN | 3.9 |
| QNLHAIHTLAIRYAN | -0.7 |
| QMHHAIHTLAIRYAN | -5 |
| QGLPAIHTLAIRYAN | 9 |
| QSHHAIHTLAIRYAN | -11.4 |
| QMLHAIHTLAIRYAN | 11.1 |
| QLLHAIHTLAIRYAN | 13.6 |
| QNHHAIHTLAIRYAN | -16.8 |
| **QTLIAIHTLAIRYAN** | **28.3** |
| QMLKAIHTLAIRYAN | 11.9 |
| QNLPAIHTLAIRYAN | 4.9 |
| QTHHAIHTLAIRYAN | -10.7 |
| QTLKAIHTLAIRYAN | 6.2 |

**Table S3.** List of ranked peptides based on the average scores that were predicted with the PARCE protocol using the first design run strategy based on random mutations. The changes are made in the TLIA fragment and the flanking amino acids of P10 that form the β-sheet after 100 mutation attempts. Aggrescan values are included. The P10 peptide is in bold.

| **Peptide** | **Aggrescan** |
| --- | --- |
| QTLIAIHTLAISYAN | 35.8 |
| HTYIAIHTLAISYMN | 40.2 |
| HYYMVIHTLAIQYMC | 50.9 |
| HTYIAIHTLAISYAN | 35.1 |
| HYYIAIHTLAISYHN | 36.9 |
| HYYFAIHTLAITYMN | 47.8 |
| HYYTVIHTLAIQYMC | 42.4 |
| HYYFAIHTLAISYMN | 46.7 |
| HYYEAIHTLAISYMN | 21.3 |
| HYYMVIHTLAIQYIC | 55.8 |
| HYYFNIHTLAITYMN | 39.3 |
| HTLIAIHTLAISYAN | 36.6 |
| HYYFNIHTLAISYMN | 38.2 |
| **QTLIAIHTLAIRYAN** | **28.3** |
| HYYFVIHTLAISYMC | 65.2 |
| HYYFVIHTLAIQYMC | 57.7 |
| HYYMVIHTLAIPYIC | 63 |
| HYYMNIHTLAIQYIC | 36.5 |
| HYYIAIHTLAISYMN | 47.2 |
| HYYFNIHTLAISYMC | 45.9 |
| HYYTVIHTLAIQYIC | 47.2 |

**Table S4.** List of ranked peptides based on the average scores that were predicted with the PARCE protocol using the second design run strategy based on bioinformatics filters. The changes are made only in the TLIA fragment of P10 that forms the β-sheet after 50 mutation attempts. Aggrescan values are included. The P10 peptide is in bold.

| **Peptide** | **Aggrescan** |
| --- | --- |
| QPFCDIHTLAIRYAN | 8.1 |
| QPFVDIHTLAIRYAN | 16 |
| QTLLDIHTLAIRYAN | 12.7 |
| QPFPDIHTLAIRYAN | 0.6 |
| QPFQDIHTLAIRYAN | -6.6 |
| **QTLIAIHTLAIRYAN** | **28.3** |
| QTLIDIHTLAIRYAN | 16.3 |
| QPFPYIHTLAIRYAN | 20.5 |
| QQLLDIHTLAIRYAN | 7 |
| QSFCDIHTLAIRYAN | 8.3 |
| QSLLDIHTLAIRYAN | 12 |
| QSLCDIHTLAIRYAN | 5.8 |
| QPFPIIHTLAIRYAN | 25 |
| QGFCDIHTLAIRYAN | 7 |

**Table S5.** List of ranked peptides based on the average scores that were predicted with the PARCE protocol using the second design run strategy based on bioinformatics filters. The changes are made in the TLIA fragment and the flanking amino acids of P10 that form the β-sheet after 100 mutation attempts. Aggrescan values are included. The P10 peptide is in bold.

| **Peptide** | **Aggrescan** |
| --- | --- |
| FTFIPIHTLAIRYTW | 49.4 |
| ITLQAIHTLAIRLAN | 17.5 |
| QTLIAIHTLAIRYAC | 35.9 |
| QTLIAIHTLAIRYYC | 42.3 |
| QTLIAIHTLAIRYFN | 37.8 |
| QTLQAIHTLAIRLAN | 5.3 |
| ATLIAIHTLAIRYFN | 42.6 |
| FTLIAIHTLAIRYYW | 55.9 |
| QTTISIHTLAIRYAN | 16.3 |
| FTFIPIHTLAIRYYW | 56.4 |
| FTLIAIHTLAIRYYC | 54.2 |
| QTLTAIHTLAIRYAN | 12.4 |
| PTGIHIHTLAIRYFN | 22 |
| MTGRHIHTLAIRYFN | 2.5 |
| QTLISIHTLAIRYAN | 26.5 |
| MTGDHIHTLAIRYFN | -2.3 |
| IGLQYIHTLAIRMAN | 20.3 |
| ITLQYIHTLAIRLAN | 25.5 |
| **QTLIAIHTLAIRYAN** | **28.3** |
| ATGIAIHTLAIRYFN | 29.8 |
| ITLQYIHTLAIRMAN | 22.3 |
| FTFIAIHTLAIRYYW | 58.4 |
| MTGIHIHTLAIRYFN | 27 |
| QTLIAIHTLAIRMAN | 26.6 |
| QTLIAIHTLAIRLAN | 29.7 |
| PTGIAIHTLAIRYFN | 28.6 |
| ITLIAIHTLAIRYYC | 54.5 |

**Supplementary Figures**


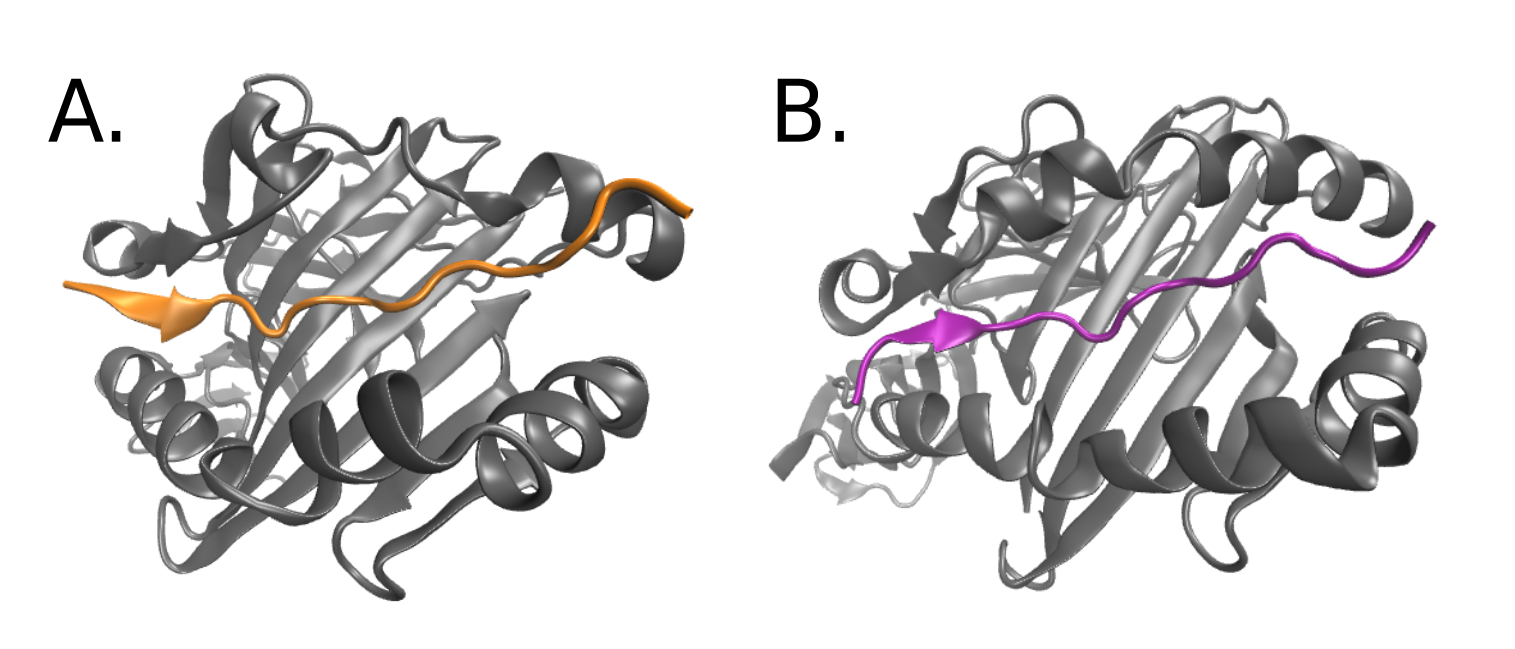


**Figure S1.** Conformations of MHC class II bound to modeled peptides after an MD simulation of 200 ns. (A) Structure of allele DRB1*03:01 (PDB id 1a6a) of MHC class II bound to a peptide template that was replaced by the P10 peptide (orange). The β-sheet region is formed at the N-terminal part of the P10 sequence. (B) Structure of allele DRB1*01:01 (PDB id 1t5x) bound to the original crystallized peptide, but with the TLIA fragment inserted in the N-terminal region, generating the sequence AA**TLIA**ATPLLLSPR (purple). As in the previous case, the β-sheet is also formed after the simulation.


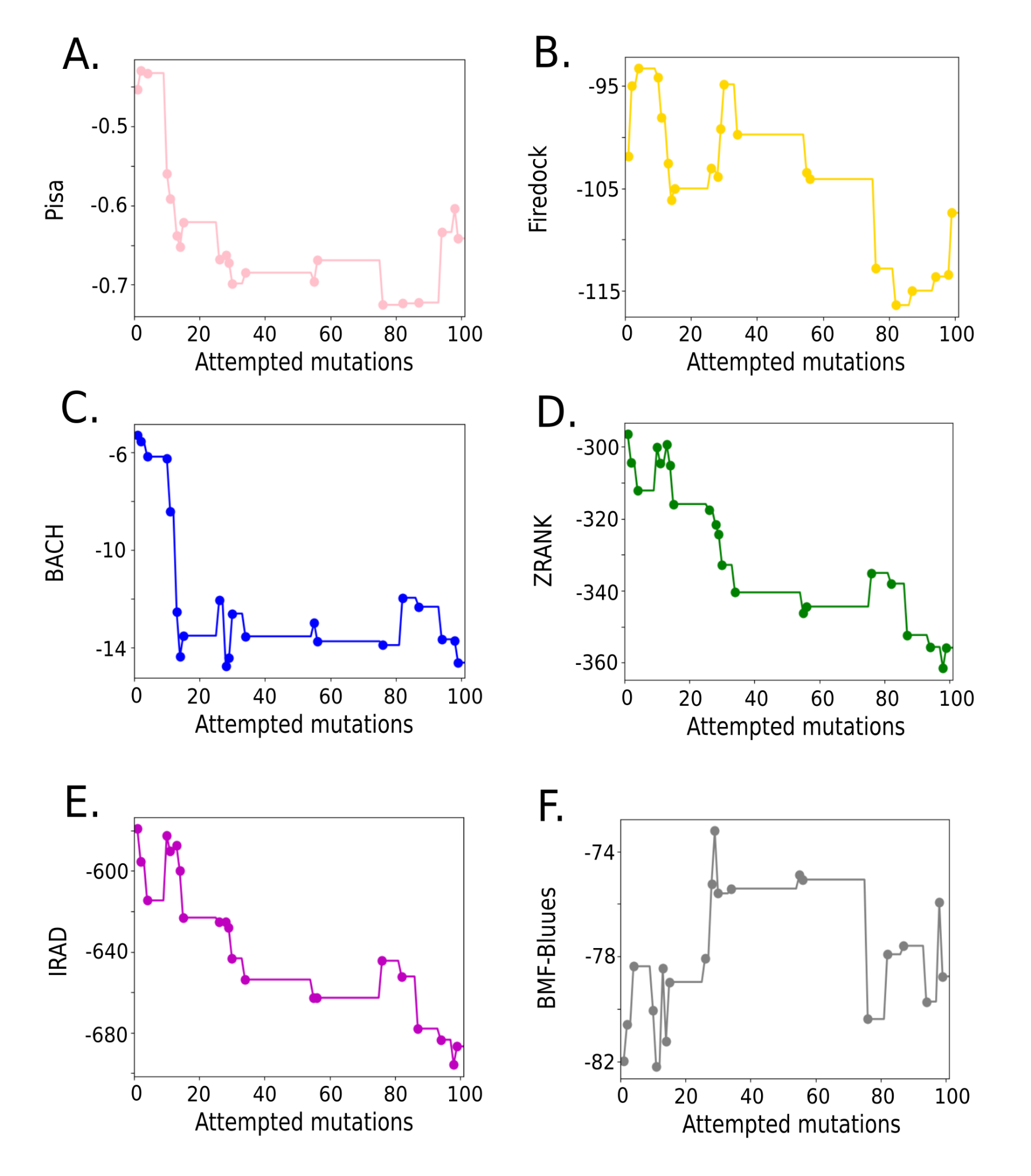


**Figure S2.** PARCE score evolution for the design run 2 using the TLIA fragment and flanking amino acids of P10. A total of 100 mutation attempts using the six scoring functions: (A) Pisa, (B) Firedock, (C) BACH, (D) ZRANK, (E) IRAD and (F) BMF-BLUUES.


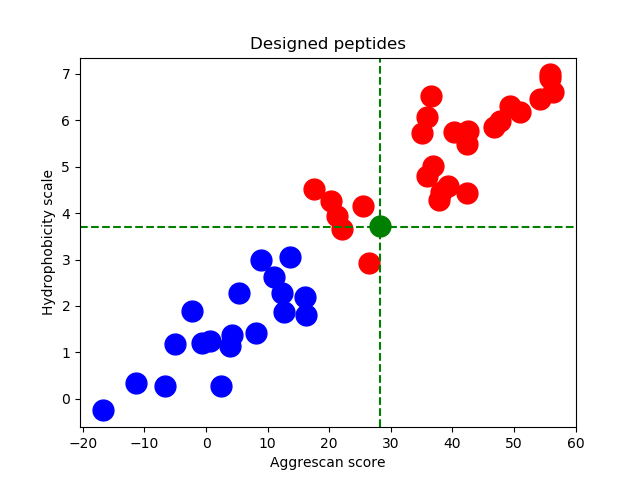


**Figure S3.** Plot showing the 46 peptides designed with all the PARCE strategies. In the X-axis is the aggregation score and in the Y-axis the hydrophobicity scale. The red circles are the peptides discarded based on worse properties than P10 (green circle). The blue circles are the 19 peptides selected to run longer MD simulations.


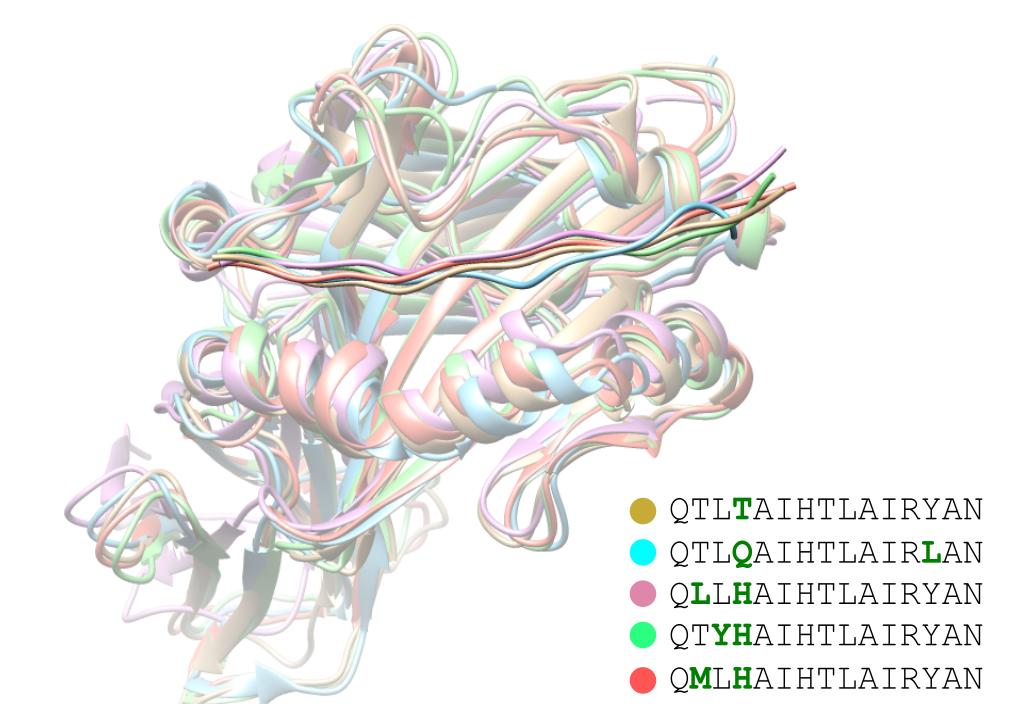
**Figure S4.** Overlapping of some of the peptide variants after running the long MD simulations of 100 ns. The variations with respect to the P10 sequence are shown in green.


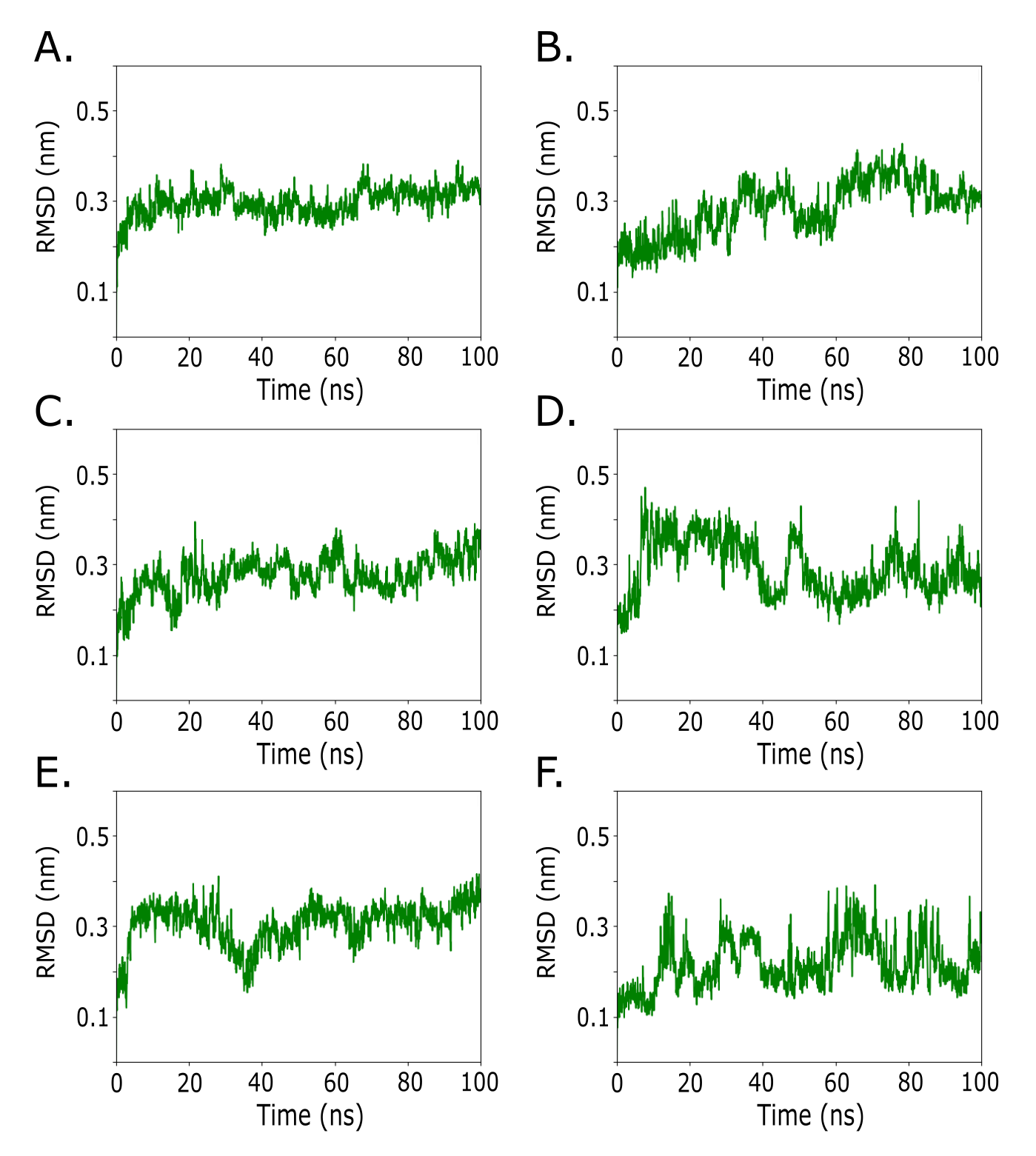


**Figure S5.** RMSD curves for the designed peptides during the 100 ns MD simulations. The sequences and internal codes are (A) QTLLDIHTLAIRYAN - V1, (B) QNLHAIHTLAIRYAN - V2, (C) QPFPDIHTLAIRYAN - V3, (D) QPFCDIHTLAIRYAN - V4, (E) QPFQDIHTLAIRYAN - V5 and (F) QTLTAIHTLAIRYAN - V6.


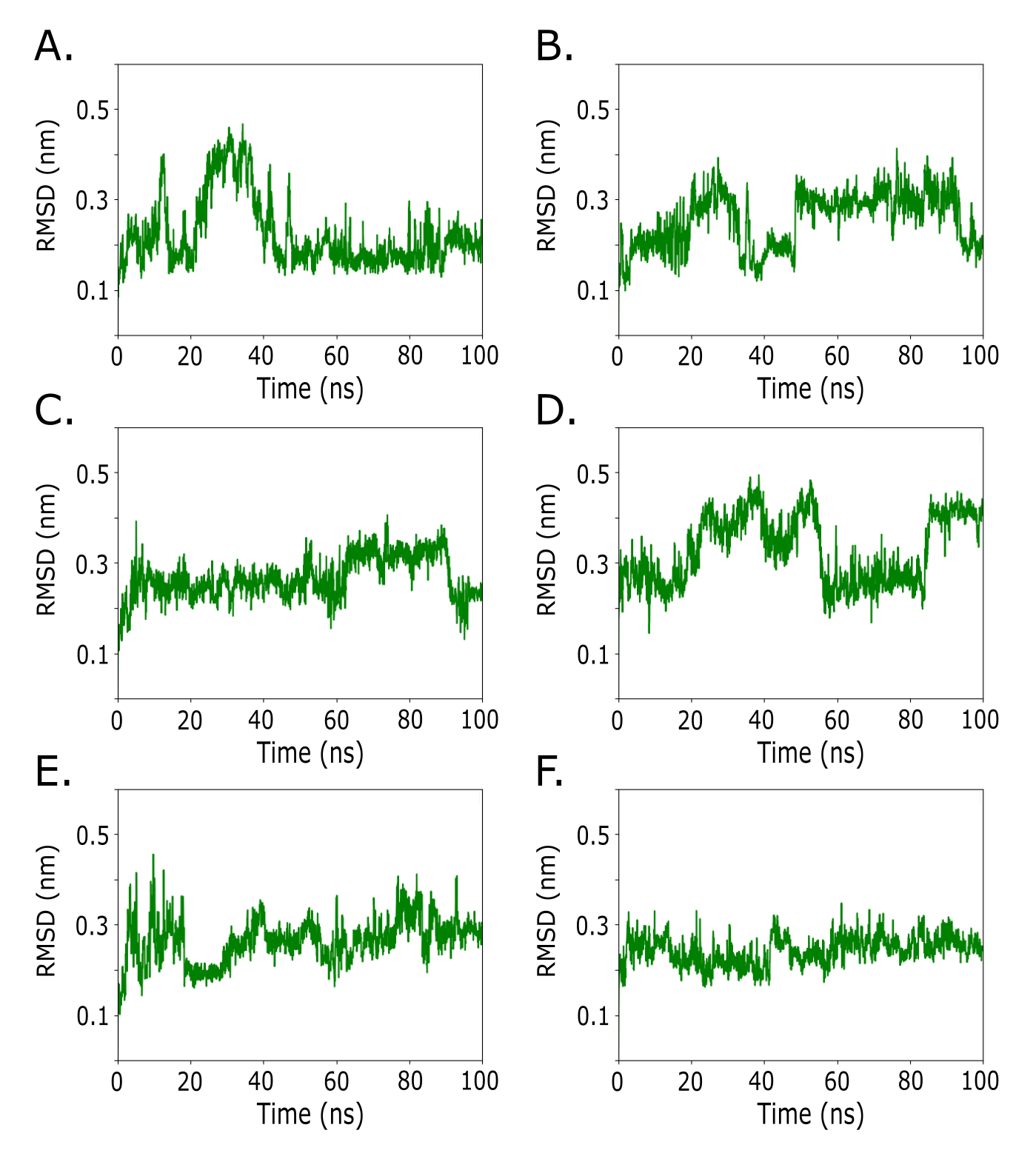


**Figure S6.** RMSD curves for the designed peptides during the 100 ns MD simulations. The sequences and internal codes are (A) QGLPAIHTLAIRYAN - V7, (B) QMHHAIHTLAIRYAN - V8, (C) QPFVDIHTLAIRYAN - V9, (D) QGLKAIHTLAIRYAN - V10, (E) QTLQAIHTLAIRLAN - V11 and (F) QSHHAIHTLAIRYAN - V12.


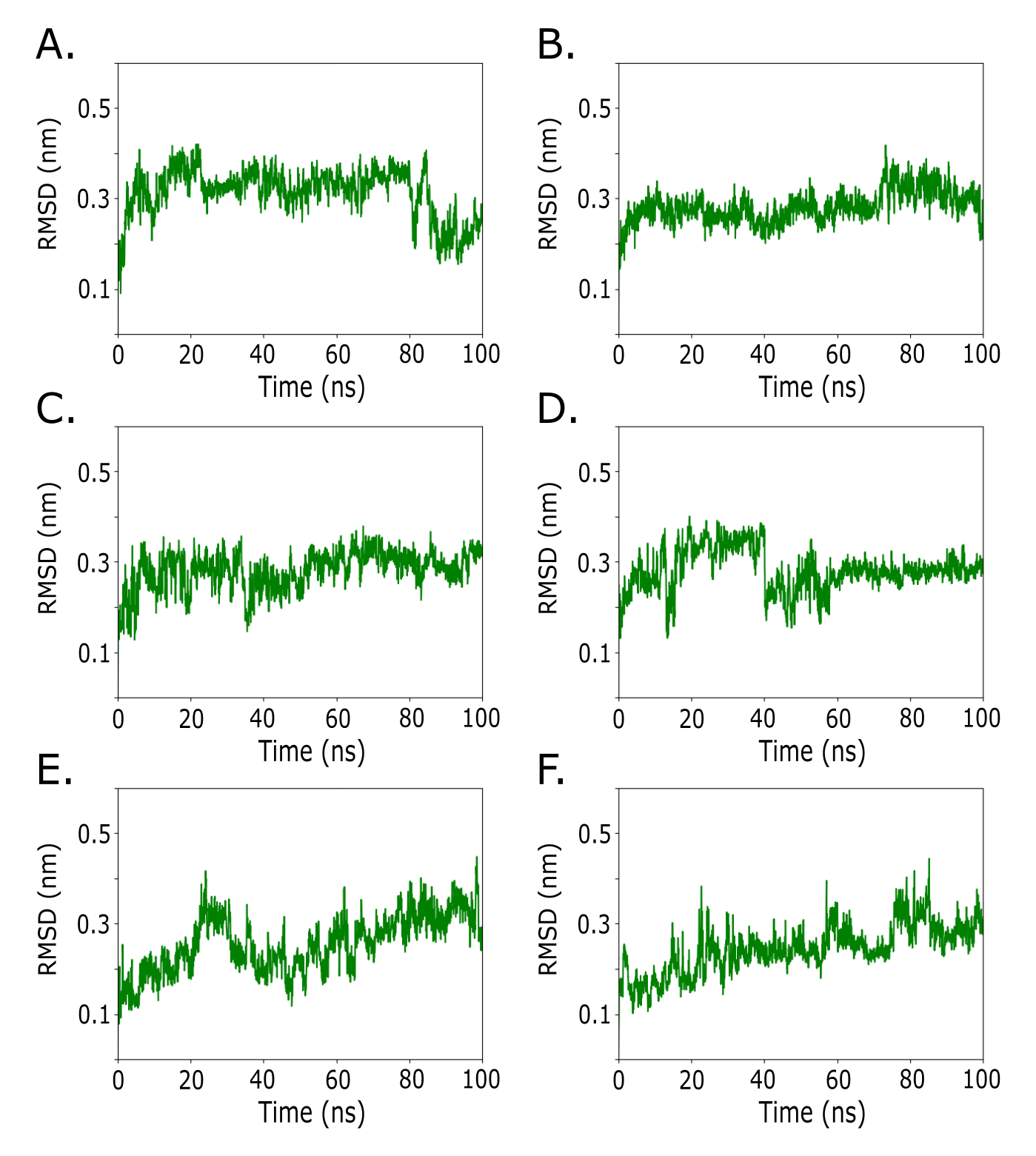


**Figure S7.** RMSD curves for the designed peptides during the 100 ns MD simulations. The sequences and internal codes are (A) QLLHAIHTLAIRYAN - V13, (B) QTYHAIHTLAIRYAN - V14, (C) QNHHAIHTLAIRYAN - V15, (D) QMLHAIHTLAIRYAN - V16, (E) QTTISIHTLAIRYAN - V17 and (F) MTGDHIHTLAIRYFN - V18.

**Supplementary Notes**

**Supplementary Note 1: Main steps of the design protocol**

**Mutation:** After sampling with MD the initial structure, we used the last frame and generated single-point mutations on the peptide sequence following different strategies. These included random selection of amino acids and prioritization of residues based on bioinformatics filters (see more details in the next subsection). The prediction of rotamers for the mutated amino acids was done with Scwrl4 [[1]](https://paperpile.com/c/Mw3W7H/PU9sD). The program was selected based on a previous study that assessed if a mutation protocol can predict amino acid rotamers similar to rotamers frequently explored by MD conformations [[2]](https://paperpile.com/c/Mw3W7H/R0ngQ). After performing the mutation, a first minimization of the predicted side chain alone is performed. In order to relocate overlapping atoms and avoid clashes, a second minimization is run with the new amino acid and the water molecules surrounding it within 2 Å. Finally, a minimization of the full system is performed with a subsequent NVT equilibration of 100 picoseconds (ps). Then, the new system is sampled for 5 ns using the MD setup explained in the previous section, and a new mutation is performed following the same rules.

**Scoring and consensus strategy:** After performing the mutated peptide-protein complex simulations, the conformations of the trajectories were scored with a chosen set of scoring functions used for protein-protein and protein-peptide affinity predictions. We implemented six scoring functions: BACH [[3,4]](https://paperpile.com/c/Mw3W7H/cI5A+ZqGZ), ZRANK [[5]](https://paperpile.com/c/Mw3W7H/weN0), IRAD [[6]](https://paperpile.com/c/Mw3W7H/hht9), Pisa [[7]](https://paperpile.com/c/Mw3W7H/s7gn), FireDock [[8]](https://paperpile.com/c/Mw3W7H/x51X), and the BMF-BLUUES scoring combination [[9,10]](https://paperpile.com/c/Mw3W7H/aaDF+oNpl). Each snapshot of the trajectory was scored, and a final average for each scoring function was used to implement the consensus strategy. Details of each scoring function are provided in the Supplementary Note 2.

The mutation is accepted following a consensus-based approach with the chosen *N* scoring functions. If a particular number *n* of scoring functions agrees with negative scoring differences between peptide *A* (original) and peptide *B* (mutated), then the final consensus will accept the change between the peptides. Based on previous studies, a threshold of 3 (from 6 scoring functions), was defined to accept or reject the mutations.

**Ranking:** From the list of peptide sequences accepted by the different strategies, we used the scores calculated from the 5 ns MD simulations to obtain an average rank per each peptide. Specifically, all the accepted peptides were ranked using each scoring function, and the average rank over the six functions was used to prioritize those peptides with potentially better affinity. After having a score-based rank of all accepted peptides, the three bioinformatics variables were calculated (see Supplementary Note 3).

**Supplementary Note 2**

The scoring functions used in this study are mostly statistical and knowledge-based potentials used for protein-protein and protein-ligand docking. We also include some semi-empirical approaches. All the scoring functions used in this work are open source. In the following, we describe the general details about their foundations.

- Pisa (version 2011): Scoring function with an all-atom potential description. It is dependent on the interface area, the residue/atom composition and contacts, hydropathy index, charge distribution, topological complementarity, and other parameters. One of its main applications is to score crystal packing of biologically relevant structures. This scoring function is commonly used with PIE to rescore conformations, but in this work was used alone with the default parameters [[7]](https://paperpile.com/c/Mw3W7H/s7gn).
- BMF (version 3): Set of empirical energies calculated using statistical information from ensembles of protein structures, reflecting the propensity of each amino acid to interact with others. The methodology has been applied for protein-fold recognition problems, and combined with other functions such as Bluues (as applied in this work) [[9]](https://paperpile.com/c/Mw3W7H/aaDF).
- Bluues (version 2): The method was developed to run analysis of electrostatic properties of proteins. It calculates the generalized Born radii, the electrostatic solvation free energy, the electrostatic forces on each atom, pH-dependent properties, pKa of all ionizable groups, and the electrostatic potentials at the surface of the molecules and in a volume surrounding it. The methodology has been combined with other functions such as BMF (as applied in this work) [[10]](https://paperpile.com/c/Mw3W7H/oNpl).
- BMF-Bluues: The arithmetic combination of the BMF and Bluues outputs.
- Firedock: Docking program with a physical scoring function based on the estimation of binding free energies, solvation, electrostatics, van der Waals, hydrogen bonds, pi-stackings, rotamer torsion energies, aliphatic interactions, and the degree of exposure of the residues. The weights associated with each term are optimized using a machine learning approach [[8]](https://paperpile.com/c/Mw3W7H/x51X).
- IRAD: Update of the original ZRANK scoring function. The new version includes an additional four residue-based potentials, where three of them account for protein-protein interactions, and one for protein folding. The implementation was applied with the complete peptide-protein complex [[6]](https://paperpile.com/c/Mw3W7H/hht9).
- ZRANK: Scoring function designed to rank structures predicted by the docking program ZDOCK. This considers a linear combination of atom energy terms weighted by optimized parameters. The terms include van der Waals attractive and repulsive terms, electrostatic attractive and repulsive, long- and short-range terms and an additional potential derived from monomeric protein structures [[5]](https://paperpile.com/c/Mw3W7H/weN0).
- BACH (version 6): Residue-wise knowledge-based potential that implements a Bayesian formalism, where the contributions account for interactions between amino acids and protein-solvent interactions. The contacts between amino acids include secondary structure specific contacts, Van der Waals contacts, among others. The program was run considering only the residues located at the interface between the peptide and receptor [[3,4]](https://paperpile.com/c/Mw3W7H/cI5A+ZqGZ).

**Supplementary Note 3: Empirical rules for peptide solubility and synthesis filtering**

Three filters were applied in addition to the consensus criteria. Two consisted of empirical rules to account for solubility and synthesis issues associated with peptides. The rules describe violations raised by certain patterns or amino acids found in the peptide sequence [[11]](https://paperpile.com/c/Mw3W7H/cqLf). The higher the number of violations, the lower the probability to validate the peptides experimentally. The solubility rules violations are:

- Discard if the number of charged and/or of hydrophobic amino acids exceeds 45%
- Discard if the absolute total peptide charge at pH 7 is more than +1
- Discard if the number of glycine or proline is more than one in the sequence
- Discard if the first or the last amino acid is charged
- Discard if any amino acid represents more than 25% of the total sequence

The synthesis rules violations are:

- Discard if 2 prolines are consecutive
- Discard if the motifs DG and DP are present in the sequence - 2 rules, one per motif
- Discard if the sequences end with N or Q residues
- Discard if there are charged residues every 5 amino acids
- Discard if there are oxidation-sensitive amino acids (M, C or W) - 3 rules, one per amino acid.

The third filter was the calculation of a peptide-hydrophobic score using the Eisenberg hydrophobicity scale defined for proteinogenic amino acids [[12]](https://paperpile.com/c/Mw3W7H/Bccd), where hydrophobic scores over 3 were discarded.

**References**

[1] [Peterson LX, Kang X, Kihara D. Assessment of protein side-chain conformation prediction methods in different residue environments. Proteins 2014;82:1971–84.](http://paperpile.com/b/Mw3W7H/PU9sD)

[2] [Ochoa R, Soler MA, Laio A, Cossio P. Assessing the capability of in silico mutation protocols for predicting the finite temperature conformation of amino acids. Phys Chem Chem Phys 2018;20:25901–9.](http://paperpile.com/b/Mw3W7H/R0ngQ)

[3] [Sarti E, Granata D, Seno F, Trovato A, Laio A. Native fold and docking pose discrimination by the same residue-based scoring function. Proteins 2015;83:621–30.](http://paperpile.com/b/Mw3W7H/cI5A)

[4] Sarti E, Zamuner S, Cossio P, Laio A, Seno F, Trovato A. [BACHSCORE. A tool for evaluating efficiently and reliably the quality of large sets of protein structures. Comput Phys Commun 2013;184:2860–5.](http://paperpile.com/b/Mw3W7H/ZqGZ)

[5] [Pierce B, Weng Z. ZRANK: reranking protein docking predictions with an optimized energy function. Proteins 2007;67:1078–86.](http://paperpile.com/b/Mw3W7H/weN0)

[6] [Vreven T, Hwang H, Weng Z. Integrating atom-based and residue-based scoring functions for protein-protein docking. Protein Sci 2011;20:1576–86.](http://paperpile.com/b/Mw3W7H/hht9)

[7] [Krissinel E, Henrick K. Inference of macromolecular assemblies from crystalline state. J Mol Biol 2007;372:774–97.](http://paperpile.com/b/Mw3W7H/s7gn)

[8] [Andrusier N, Nussinov R, Wolfson HJ. FireDock: fast interaction refinement in molecular docking. Proteins 2007;69:139–59.](http://paperpile.com/b/Mw3W7H/x51X)

[9] [Berrera M, Molinari H, Fogolari F. Amino acid empirical contact energy definitions for fold recognition in the space of contact maps. BMC Bioinformatics 2003;4:8.](http://paperpile.com/b/Mw3W7H/aaDF)

[10] [Fogolari F, Corazza A, Yarra V, Jalaru A, Viglino P, Esposito G. Bluues: a program for the analysis of the electrostatic properties of proteins based on generalized Born radii. BMC Bioinformatics 2012;13 Suppl 4:S18.](http://paperpile.com/b/Mw3W7H/oNpl)

[11] [Santos GB, Ganesan A, Emery FS. Oral Administration of Peptide-Based Drugs: Beyond Lipinski’s Rule. ChemMedChem 2016;11:2245–51. https://doi.org/](http://paperpile.com/b/Mw3W7H/cqLf)[10.1002/cmdc.201600288](http://dx.doi.org/10.1002/cmdc.201600288)[.](http://paperpile.com/b/Mw3W7H/cqLf)

[12] [Eisenberg D, Weiss RM, Terwilliger TC. The hydrophobic moment detects periodicity in protein hydrophobicity. Proc Natl Acad Sci U S A 1984;81:140–4.](http://paperpile.com/b/Mw3W7H/Bccd)
